# Reducing unnecessary biopsies of BI-RADS 4 lesions based on a deep learning model for mammography

**DOI:** 10.3389/fonc.2025.1543553

**Published:** 2025-06-03

**Authors:** Yuting Yang, Tingting Liao, Xiaohui Lin, Rushan Ouyang, Zhenjie Cao, Jingtao Hu, Jie Ma

**Affiliations:** ^1^ Shenzhen People’s Hospital, The Second Clinical Medical College of Jinan University, Shenzhen, China; ^2^ Department of Radiology, The Eighth Affiliated Hospital, Sun Yat-sen University, Shenzhen, China; ^3^ Shenzhen International Graduate School, Tsinghua University, Shenzhen, Guangdong, China; ^4^ Department of Pathology, Shenzhen People’s Hospital, Shenzhen, China; ^5^ Department of Anatomical and Cellular Pathology, Prince of Wales Hospital, The Chinese University of Hong Kong, Hongkong, Hong Kong SAR, China

**Keywords:** mammography, artificial intelligence, deep learning, breast cancer, BI-RADS 4 lesions

## Abstract

**Objective:**

In this study, we aimed to explore the diagnostic value of a deep learning (DL) model based on mammography for Breast Imaging Reporting and Data System (BI-RADS) 4 lesions and to reduce unnecessary breast biopsies.

**Methods:**

We retrospectively collected clinical and imaging data of 557 BI-RADS 4 lesions (304 benign lesions, 195 malignant lesions, and 58 high-risk lesions which have risk of developing malignancy) obtained by mammography at Shenzhen People’s Hospital and Luohu People’s Hospital from January 2020 to June 2022. The DL model was constructed to predict the pathological classifications of these lesions, calculated its sensitivity, specificity, and accuracy, and evaluated its diagnostic performance using receiver operating characteristic curve and area under the curve (AUC).

**Results:**

This study included 557 patients with BI-RADS 4 lesions, including 381 patients (68.40%) with BI-RADS 4A, 106 patients (19.03%) with BI-RADS 4B, and 70 patients (12.57%) with BI-RADS 4C. For BI-RADS categories 4A, 4B, and 4C lesions, 70.9%, 27.4%, and 7.1% were respectively confirmed as benign through biopsy, surgical pathology, or follow-up. The DL model demonstrated high diagnostic performance in identifying BI-RADS 4 lesions, achieving a sensitivity of 81.0%, specificity of 76.9%, accuracy of 78.8%, and an AUC of 0.790. We found that our DL model could avoid unnecessary biopsies for BI-RADS 4 lesions by 40.6% in our included patients, reducing unnecessary biopsies for BI-RADS 4A, 4B, and 4C lesions by 55.1%, 18.9%, and 4.29%, respectively.

**Conclusion:**

Our DL model for classifying BI-RADS 4 lesions can accurately identify benign and high-risk lesions that do not necessitate biopsy, further enhancing the safety and convenience for patients.

## Introduction

According to the latest global cancer data released by the World Health Organization in 2024, there were ~2.3 million new cases of breast cancer worldwide in 2022 (1). Mammography is the only imaging method approved by the U.S. Food and Drug Administration (FDA) for effective and early detection of lesions and consequently reducing the mortality rate of breast cancer. However, the detection of cancerous lesions with mammography can be challenging in dense breasts, as the high density of surrounding noncancerous tissue may obscure these lesions. This reduces their visibility, leading to decreased sensitivity in mammography and a higher rate of false positives, which can result in lesions being underdiagnosed or misdiagnosed (2).

According to the Breast Imaging Reporting and Data System (BI-RADS) of the American College of Radiology (5th edition), the possibility of malignancy in a lesion classified as BI-RADS 4 is 2%–95%, which is further subdivided into 4A (possibility of malignancy: >2% to ≤10%), 4B (possibility of malignancy: >10% to ≤50%), and 4C (possibility of malignancy: >50% to <95%), and it is recommended that biopsy be performed for BI-RADS 4 lesions if there are no clinical contraindications (3). In clinical practice, owing to the lack of objective criteria for subcategorizing BI-RADS 4 lesions, the diagnosis of BI-RADS 4 lesions is largely determined by the clinical experience and subjectivity of radiologists, which leads to a large proportion of patients undergoing unnecessary biopsies and waste of medical resources. Research has indicated that 70%–80% of biopsies performed on suspicious breast lesions were confirmed to be benign (4, 5). Given the situation, effective and accurate adjunctive diagnostic methods are urgently needed to prevent patients from unnecessary biopsies and circumvent waste of medical resources.

With the rapid development of deep learning (DL) algorithms in recent years, significant achievements have been made in medical image analysis across multiple fields (6). DL can automatically extract image features, offering new opportunities in early detection, accurate diagnosis, and personalized treatment of breast cancer (7). Numerous studies have confirmed that DL can help radiologists improve work efficiency and diagnostic efficiency and reduce missed diagnosis of lesions (8, 9). Despite the advancements in DL, relatively few studies have specifically examined its role in mammography for BI-RADS 4 lesions. Consequently, the primary aim of this study was to explore the diagnostic effectiveness of DL models in predicting BI-RADS 4 lesions and to evaluate their potential in reducing unnecessary biopsies of benign breast lesions.

## Materials and methods

### Enrollment of patients

**Figure 1:**
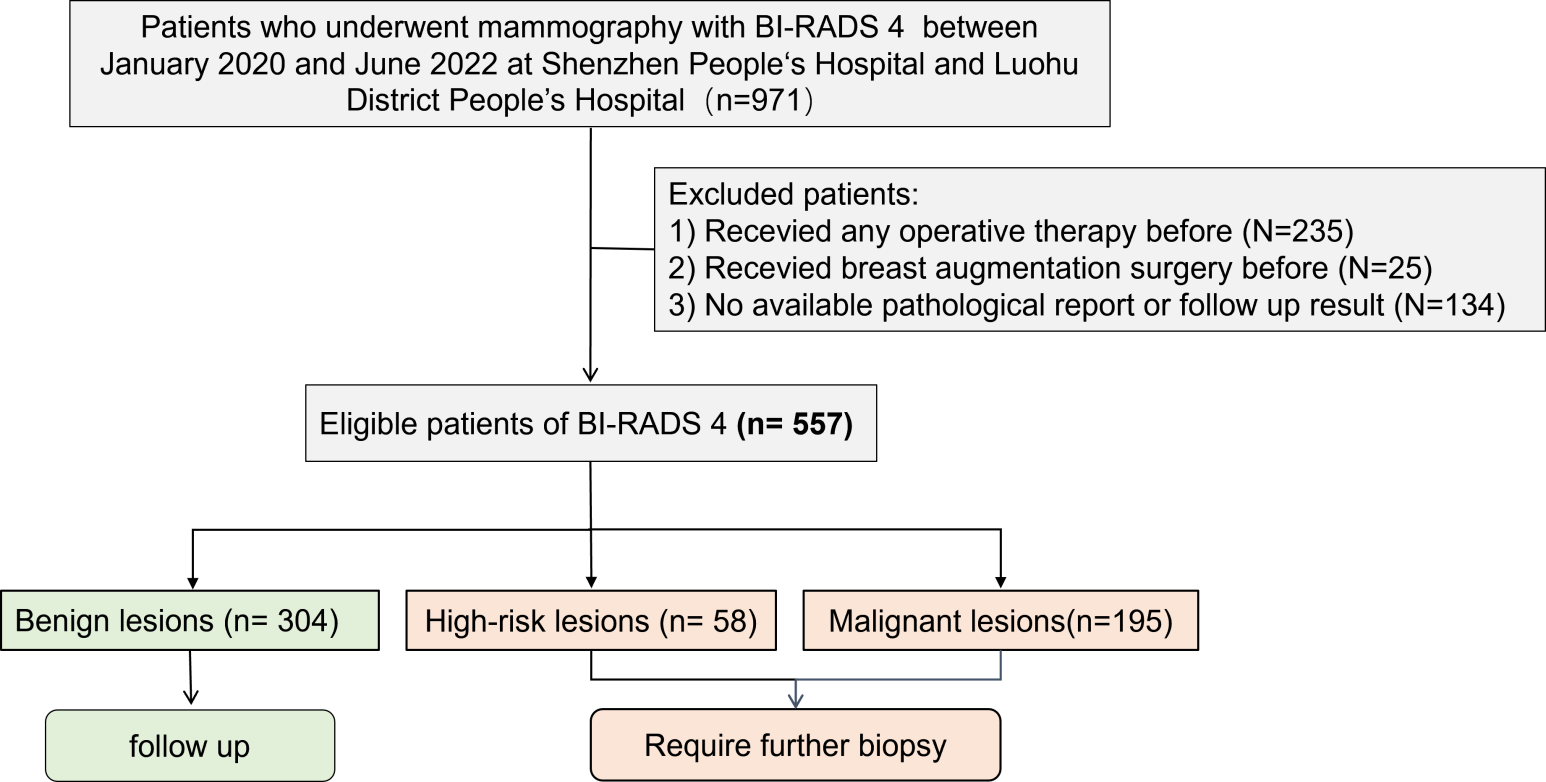
Patient screening flowchart. This study included a retrospective collection of clinical, pathologic, and imaging data of 557 patients with breast lesions assessed as BI-RADS 4 by mammography at Shenzhen People’s Hospital and Luohu People’s Hospital from January 2020 to June 2022. After excluding 223 patients with a history of surgery, 131 patients with no pathological results, 28 patients without complete follow-up results for at least 2 years, and 12 patients with a history of breast augmentation, a total of 557 patients were finally included. Among them, there were 304 cases of benign lesions, 58 cases of high-risk lesions, 195 cases of malignant lesions.

This study included a retrospective collection of clinical, pathologic, and imaging data of 557 patients with breast lesions assessed as BI-RADS 4 by mammography at Shenzhen People’s Hospital and Luohu People’s Hospital from January 2020 to June 2022 (**Figure 1**). Clinical data were collected for age, menopausal status, family history of breast cancer, and clinical manifestations. The inclusion criteria were as follows: (1) diagnostic mammography report assessed as BI-RADS 4 (4A, 4B, or 4C); (2) availability of complete bilateral craniocaudal (CC) and mediolateral oblique (MLO) images; and (3) availability of at least 2 years of follow-up data, including needle biopsy or surgical pathology findings. The exclusion criteria were as follows: (1) the image quality not meeting diagnostic requirements and (2) previous history of breast augmentation, surgery, or trauma on either breast. Data of a total of 971 patients were initially collected in this study; however, after excluding 223 patients with a history of surgery, 131 patients with no pathological results, 28 patients without complete follow-up results for at least 2 years, and 12 patients with a history of breast augmentation, a total of 557 patients [age, 47.6 ± 10.1 years] were finally included. This study was approved by the Ethics Committee of Shenzhen People’s Hospital (LL-KY-2021624).

### Mammography examination

Mammography examination was performed using the Mammomat Inspiration (Siemens Healthineers, Forchheim, Germany) and the MD full-field digital mammography system (Giotto Image, Sasso Marconi, Italian). All patients underwent standard bilateral CC and MLO examinations.

### Construction of DL models

**Figure 2:**
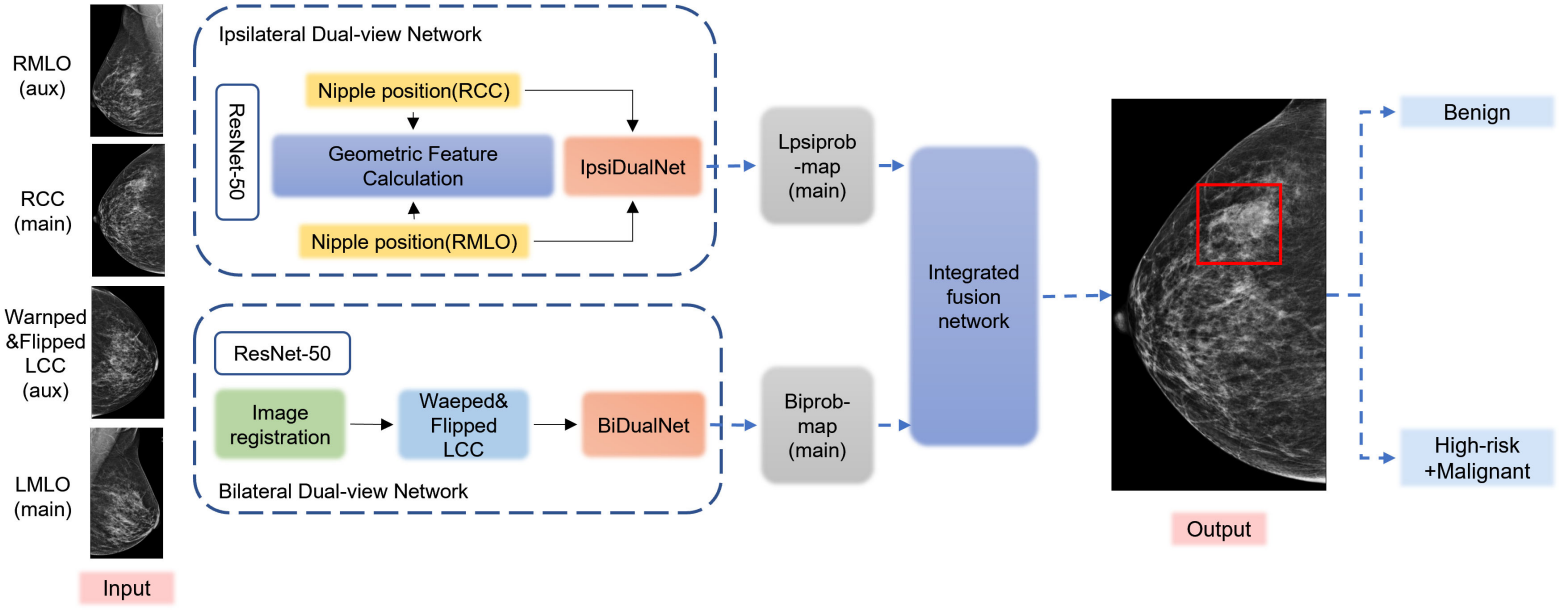
Flowchart of the structure of the deep learning model. The preprocessed DICOM images are input into the Mammo AI V3 model, which performs joint lesion detection through both calcification detection and non-calcification detection models. The system identifies lesions, extracts lesion features, and ultimately outputs classification results.

The DL model (Mammo AI V3) used in this study is a fusion deep learning model developed by Shenzhen People’s Hospital, which can achieve lesion detection, segmentation, and benign/malignant classification. This study used a deep learning-based breast X-ray lesion recognition and classification model from previous research (10). The pre trained database included 1776 cases from the internal database of Shenzhen People’s Hospital, randomly divided in an 8:1:1 ratio. The model was trained on a carefully curated dataset of 1,776 cases that underwent rigorous preprocessing including DICOM windowing, breast region segmentation, artifact removal, and random 400×400 patch extraction, with additional data augmentation through rotation (± 15°), elastic deformation (σ=8, α=20), and intensity variation (± 10%). Our training protocol employed the AdamW optimizer (β1 = 0.9, β2 = 0.999) with an initial learning rate of 3e-4 using cosine decay, with batch sizes of 32 for calcification and 16 for non-calcification tasks, and implemented a combination of specialized loss functions including Dice (0.6) + Focal (γ=2) for segmentation, modified GIoU for detection, and ordinal cross-entropy with biopsy consistency penalty for classification, along with dropout (0.3) and weight decay (1e-4) for regularization. The dataset was strategically split into training/validation/test sets using 3:1:1 ratios for the calcification and classification models and 8:1:1 for the non-calcification model (all patient-wise), with validation performed through 5-fold cross-validation during development featuring early stopping (patience=8 epochs) and model selection based on Dice coefficient for segmentation and AUC-ROC for classification, while final performance was rigorously evaluated on a completely held-out independent test set. The model includes both calcification and non-calcification detection models. The calcification detection model is an improved segmentation model based on the medical image segmentation network U-net, whereas the non-calcification detection model contains three modules: ipsilateral dual-view network (IDVN), bilateral dual-view network (BDVN), and integrated fusion network (IFN) (**Figure 2**). Notably, non-calcification detection model is capable of processing images from multiple projection angles for the same patient, utilizing two distinct high-resolution networks specifically designed for detection and segmentation of ipsilateral and contralateral images, respectively. This helps jointly detect the lesions through the nipple detection algorithm in combination with the target detection algorithm, which applies Densely Connected Convolutional Networks-121 (DenseNet-121). These algorithms were used to train the DL algorithm to extract lesion features and to be able to take into account the association of the BI-RADS class with benign and malignant labels.

### Statistical analysis

SPSS statistical software (Version 26.0. IBM Corp., Armonk, NY, USA) was used for data analysis. The age of patients was expressed as mean ± standard deviation (x¯ ± s), and one-way analysis of variance (ANOVA) was used to analyze the variance between groups. The chi-square test was used to compare the frequencies, and the difference was considered statistically significant at *P* < 0.05. The sensitivity, specificity, and accuracy of the DL model were calculated. The classification diagnostic efficacy of the DL model was assessed using the receiver operator characteristic (ROC) curve and the area under the ROC curve (AUC).

## Results

### Characteristics of enrolled subjects

A total of 557 patients [age, 47.6 ± 10.1 years] evaluated as having BI-RADS 4 lesions on mammography were enrolled in this study. These included 381 patients (68.4%) with BI-RADS 4A, 106 patients (19.0%) with BI-RADS 4B, and 70 patients (12.6%) with BI-RADS 4C lesions. Among the BI-RADS 4A, 4B, and 4C lesions, 70.9% (270/381), 27.4% (29/381), and 7.1% (5/381) were confirmed to be benign lesions by biopsy and surgical pathology or follow-up, respectively.

According to the European Guidelines for Diagnosis Quality Management of Breast Cancer Screening, 5%–9.2% of biopsy pathologies are high-risk lesions with the possibility of upgrading to malignancy in subsequent surgical pathology (11–13). Therefore, in this study, high-risk and malignant lesions that require further histological biopsy were grouped into a single category, comprising 58 high-risk cases and 195 malignant cases. Benign lesions that did not require further biopsy were classified separately. Consequently, BI-RADS 4 lesions were pathologically categorized as benign, high-risk, or malignant.

Among the 557 patients with BI-RADS 4 lesions, patients with benign pathology were younger than those with high-risk or malignant pathology (*P* < 0.001), and the proportion of menopausal patients was relatively smaller in patients with benign pathology (*P* < 0.001). Notably, 476 (82.5%) patients with BI-RADS 4 lesions were negative for clinical features of palpable mass, nipple fluid/blood overflow, or nipple pain, whereas 101(17.5%) patients were positive for these clinical features. In addition, 91.4% (278/304) of patients with benign pathology were negative for these clinical features, whereas 78.3% (198/253) of patients with high-risk or malignant pathology presented with the abovementioned clinical features, and the difference was statistically significant (*P* < 0.001; **Table 1**).

**Table 1:** Correlation analysis of different pathological classifications of BI-RADS 4 types of lesions with clinical and imaging features.

Clinical and Imaging Features	Total (n = 557)	Benign (n = 304)	Malignant (n = 195)	High risk (n = 58)	Statistical value	*P* value
Age (years, mean ± SD)	47.6 ± 10.1	45.0 ± 8.8	51.4 ± 11.2	48.3 ± 8.6	F = 25.78	<0.001
**Menopausal status**					χ² = 22.97	<0.001
Premenopausal	178	72	86	20		
Postmenopausal	379	232	109	38		
**Family history**					1.12	0.571
Yes	6	2	2	2		
No	551	302	193	56		
**Breast composition**					χ² = 4.29	0.638
a	11	5	5	1		
b	61	31	25	5		
c	366	195	131	40		
d	119	73	34	12		
**Clinical feature**					70.59	<0.001*
Negative	476	278	168	30		
Palpable swelling	40	18	14	8		
Fluid/blood spills	32	2	12	18		
Soreness	9	6	1	2		
**Mammogram findings**					5.61	0.784
Masses	284	162	93	29		
Calcifications	228	116	88	24		
Architectural distortion	16	10	4	2		
Asymmetries	21	12	6	3		
Associate featured	8	4	4	0		
**BI-RADS Assessment Category**					χ² = 176.14	<.001
4A	381	270	69	42		
4B	106	29	63	14		
4C	70	5	63	2		

ANOVA, χ², Chi-square test; -, Fisher exact; *, Simulated *P* value; SD, standard deviation.

In this study, biopsy/surgical pathology results or two-year follow-up results were used as the gold standard. According to the aforementioned pathological classification of BI-RADS 4 lesions used in this study, 304 were benign, 58 were high-risk, and 195 were malignant lesions. The overall malignancy rate, which is the positive predictive value (PPV) for histopathology for BI-RADS 4 lesions, was 35.0%, and the specificity were 18.11% (69/381) for BI-RADS 4A, 59.4% (63/106) for BI-RADS 4B, and 90.0% (63/70) for BI-RADS 4C lesions. The proportion of BI-RADS 4 lesions that were downgraded to benign by the DL model was 42.0% (234/557), and the proportion of 4A, 4B, and 4C lesions correctly degraded to benign was 55.1% (210/381), 18.9% (20/106) and 4.29% (3/70), respectively. There were significant differences in the downgrading rates of 4A, 4B, and 4C, and the downgrading rate of 4A was significantly higher than that of 4B and 4C (*P* < 0.001; **Tables 1**, **2**).

**Table 2:** Results of the deep learning system for predicting different pathologic classifications of BI-RADS 4 lesions.

DL forecast results	Benign			High-risk lesions + malignant		
	4A	4B	4C	4A	4B	4C
DL benign	210	20	3	37	7	4
DL malignant	60	9	2	74	70	61
Total	270	29	5	111	77	65

DL, deep learning.

### Model effectiveness

**Figure 3:**
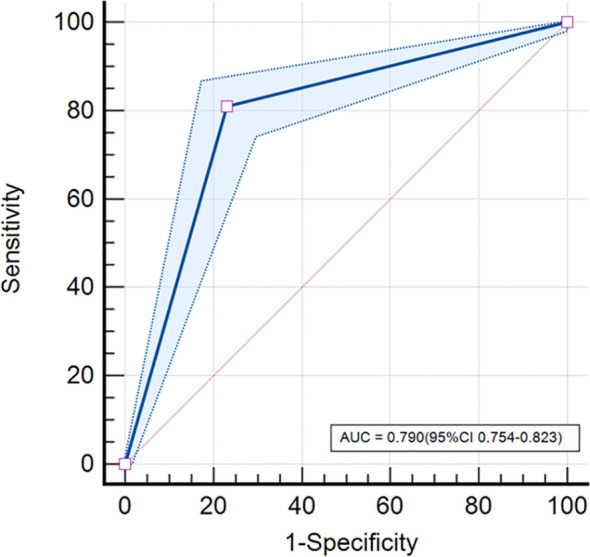
ROC curve. The DL model based on mammography achieved an AUC of 0.790 (95% confidence interval [CI]: 0.754-0.823) in classifying BI-RADS 4 lesions.

**Figure 4:**
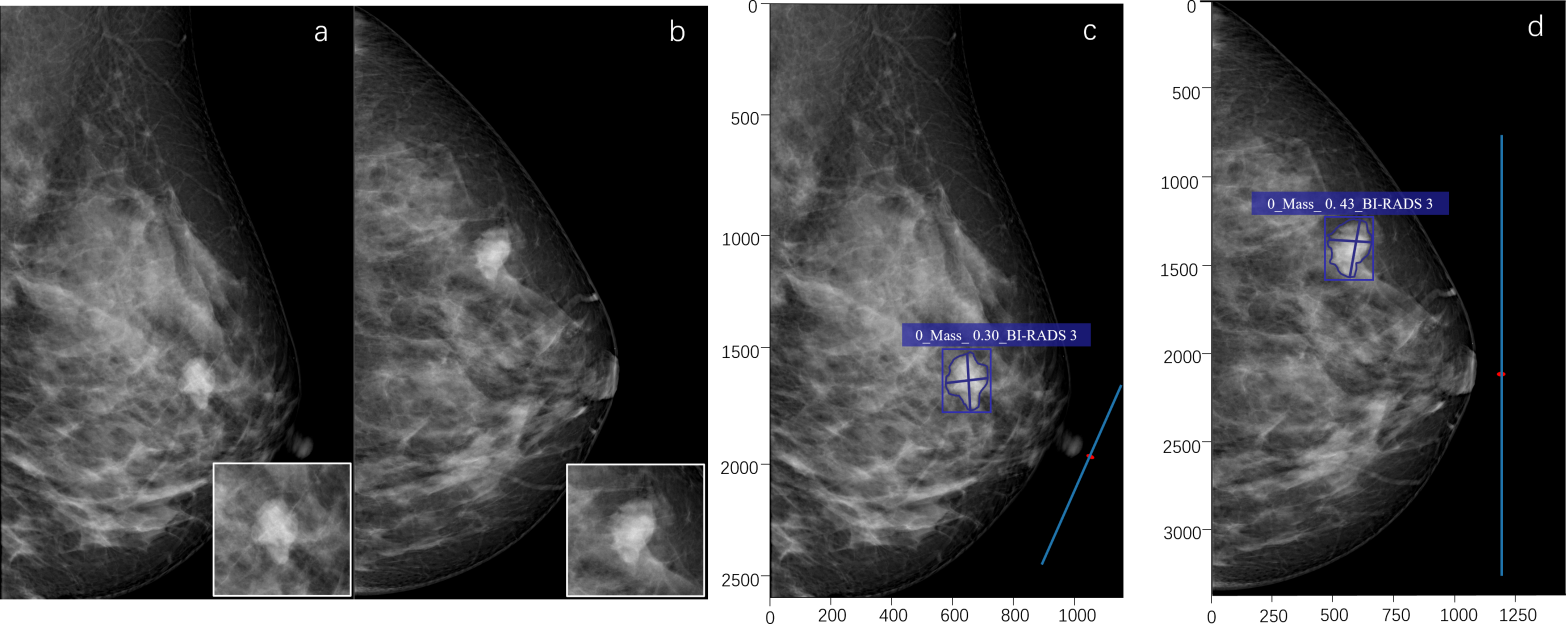
A 53-year-old female. A high-density mass with a lobulated shape is seen in the upper outer quadrant of the left breast **(a, b)**. The radiologist assessed it as BI-RADS 4B. The pathological result of puncture is breast fibroadenoma (benign). The deep learning (DL) model accurately detects the lesion, indicates that the lesion type is a mass, and the probability of malignancy is 0.30. The DL assessment is BI-RADS 3 **(c, d)**.

**Figure 5:**
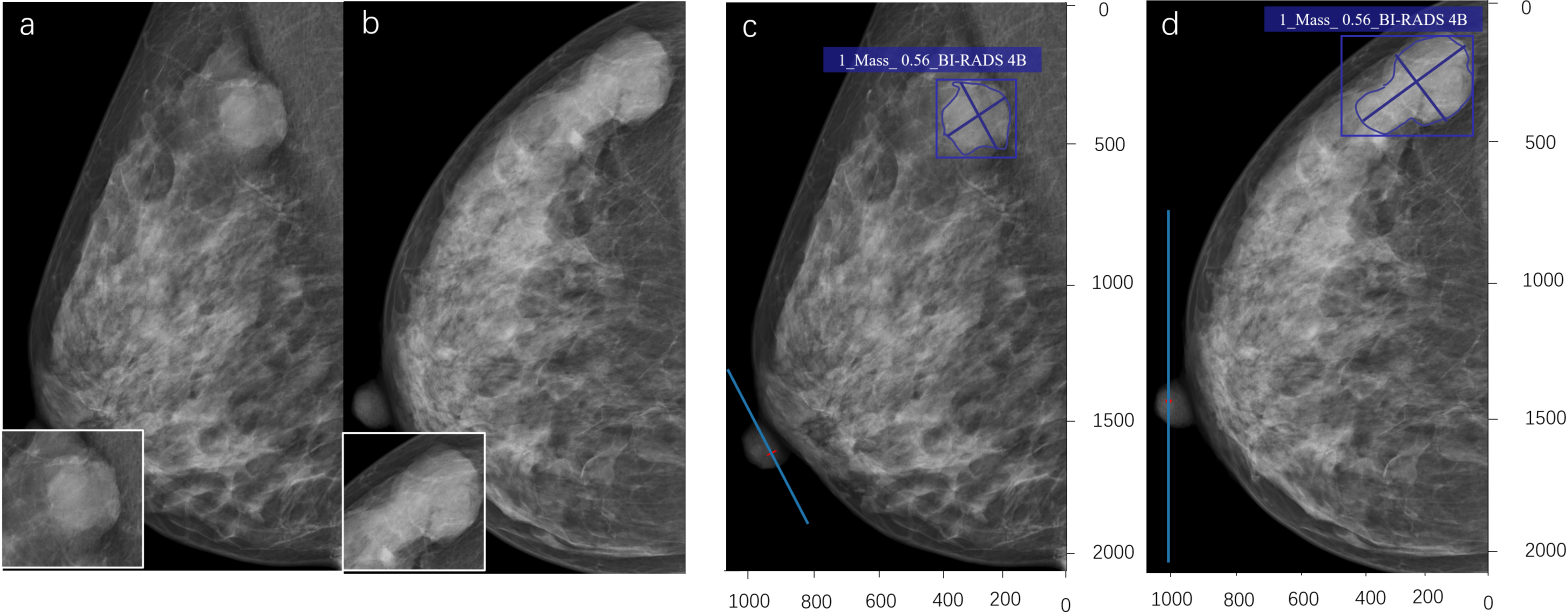
A 43-year-old woman. A shallow lobulated high-density mass can be seen in the upper outer quadrant of the right breast **(a, b)**. The radiologist evaluated it as BI-RADS 4A. The pathological result of the puncture is mucinous adenocarcinoma of the breast (malignant). The deep learning (DL) model accurately detects lesions, indicating that the lesion type is a mass, with a probability of 0.56 for malignant tumors. DL evaluation is BI-RADS 4B **(c, d)**.

Our study showed that the AUC, sensitivity, specificity, and accuracy of the DL model were 0.790, 81.0%, 76.6%, and 78.6%, respectively. The PPV and negative predictive value (NPV) were 0.743 and 0.829, respectively (**Table 3**). **Figure 3** showed the ROC curve of the DL model for the classification of BI-RADS 4 lesions. **Figures 4**, **5** provide visual examples to demonstrate that our model has good performance.

**Table 3:** Diagnostic performance of deep learning models in classifying BI-RADS 4 lesions.

Metric	Value (95% CI)
AUC	0.790 (0.754–0.823)
SEN	0.810 (0.756–0.857)
SPE	0.766 (0.715–0.813)
Accuracy	0.786 (0.750–0.820)
PPV	0.743 (0.700–0.781)
NPV	0.829 (0.789–0.863)

AUC, area under the receiver operating characteristic curve; SEN, sensitivity; SPE, specificity; PPV, positive predictive value; NPV, negative predictive value; CI, confidence interval.

## Discussion

In this study, we employed a DL model to predict the classification of BI-RADS 4 lesions. Our results demonstrated that the model exhibited strong predictive performance, achieving an AUC of 0.790 (95% CI: 0.754–0.823), a sensitivity of 81.0%, a specificity of 76.6%, and an accuracy of 78.6%. The model effectively differentiated benign from high-risk lesions, potentially eliminating the need for unnecessary biopsies.

The false-positive rate of mammography is reportedly 7%–10%, and 70%–80% of lesions assessed as BI-RADS 4 are confirmed as benign by biopsy (14); furthermore, among the BI-RADS 4 lesions, the over-diagnosis of BI-RADS 4A lesions is reportedly significantly higher than that of BI-RADS 4B and 4C lesions, with most biopsies of BI-RADS 4A lesions revealing a benign pathology (15). Our findings revealed that the PPV of BI-RADS 4 lesions was 35.01%, indicating that most patients with BI-RADS 4 lesions underwent unnecessary biopsies, with a malignancy rate of 18.11% for BI-RADS 4A (69/381), 59.4% for BI-RADS 4B (63/106), and 90.0% (63/70) for BI-RADS 4C. The PPVs of BI-RADS 4B and 4C lesions were within the specified BI-RADS class, whereas the PPV of BI-RADS 4A lesions was slightly higher than the corresponding malignant ranges (5%–10%), which could be attributed to the limited data available and variations in the subjective assessments of diagnosticians. Besides, the deep learning model in our study has a sensitivity of 0.810 and an accuracy of 0.786, with a false-negative rate of 19%. This indicates that while the model demonstrates reasonable diagnostic capability, it still misses a portion of malignant lesions, which could lead to delayed diagnosis and treatment. This may be related to the model not incorporating clinical factors.

In China, few studies have attempted to classify mammography images of BI-RADS 4 lesions using DL techniques. Chika et al. developed the iBRISK intelligent breast cancer risk calculator by combining DL techniques and clinical factors using mammography image data of 9700 patients in the United States (16). In the multicenter study, iBRISK underwent further evaluation across datasets from three medical institutions, encompassing a total of 4,209 cases. Clinically significant factors were integrated with the model, culminating in binary and ternary classification of the data to assess the probability of malignant tumors and risk stratification. Their findings revealed that the iBRISK system had an AUC of 0.93 (95% CI: 0.92–0.95) in BI-RADS 4 lesions with 89.5% accuracy and could reduce unnecessary biopsies by 50%. Its sensitivity was 100%, and specificity was 81%. Similarly, our study employed a deep learning model to investigate BI-RADS 4 lesions. The selected deep learning model, Mammo AI V3, was developed in previous research using mammographic images from 1,700 patients. Data from two institutions were evaluated in this study, encompassing 557 patients. Clinically significant factors were not incorporated in this study. Ultimately, the deep model categorized BI-RADS 4 lesions into two groups: benign and malignant or high-risk lesions, and the results revealed an AUC of 0.790 (95% CI: 0.754–0.823); the accuracy, sensitivity, and specificity were 78.8%, 81.0%, and 76.9%, respectively. Although the diagnostic efficacy was lower than that of the study of Chika et al, this may be attributed to our study not combining clinical factors, resulting in diagnostic results and clinical bias. Additionally, all the patients in our study were East Asian women, with 84.1% having dense breast tissue. This increased the diagnostic challenge due to the considerable shielding effect of the glandular tissue during mammogram lesion evaluation. Besides, the amount of data we include will also have a certain impact.

In the current research field from China, many scholars are committed to constructing DL models to improve the diagnostic efficacy of breast ultrasound images (17–19). Wang et al., after applying a deep learning model based on contrast-enhanced ultrasound to classify benign and malignant BI-RADS 4 lesions, found that the model correctly identified a greater number of BI-RADS 4 lesions as benign compared to senior doctors, without compromising diagnostic sensitivity. This approach effectively reduced the number of biopsies for false-positive lesions (18). However, there are few studies on BI-RADS 4 lesions mammography images. We found that our DL model could have reduced unnecessary biopsies for BI-RADS 4 lesions by 40.6% in our included patients; furthermore, it could have reduced unnecessary biopsies for BI-RADS 4A, 4B, and 4C lesions by 55.1%, 18.9%, and 4.29%, respectively.

This study has some limitations. First, the DL model in this study did not incorporate clinical factors like family history and clinical symptoms, which may affect the comprehensive analysis of lesions and lead to discrepancies between clinical and model-based diagnoses. Second, although the mammography images in this study were obtained from two medical institutions and from two different devices, which support the generalizability of the proposed model to some extent, prospective studies with larger sample sizes from more centers and using devices from more manufacturers are still needed to validate our model. In addition, only East Asian women are included, with a relatively small proportion of fat type glands. Therefore, in future studies, we plan to build more advanced and clinically valuable DL models using multiracial, multicenter, multimodal data and clinical information.

In summary, the DL models based on mammography facilitate the accurate assessment of breast BI-RADS 4 lesions, effectively avoiding unnecessary biopsies, reducing healthcare costs, and preventing the inconvenience and stress caused to patients.

## Data Availability

The raw data supporting the conclusions of this article will be made available by the authors, without undue reservation.
